# Sex Differences in Training Behaviors of 10 km to Ultra-Endurance Runners (Part A)—Results from the NURMI Study (Step 2)

**DOI:** 10.3390/ijerph192013238

**Published:** 2022-10-14

**Authors:** Derrick Tanous, Mohamad Motevalli, Gerold Wirnitzer, Claus Leitzmann, Thomas Rosemann, Beat Knechtle, Katharina Wirnitzer

**Affiliations:** 1 Department of Sport Science, University of Innsbruck, 6020 Innsbruck, Austria; 2 Department of Research and Development in Teacher Education, University College of Teacher Education, Tyrol, 6010 Innsbruck, Austria; 3 adventureV & change2V, 6135 Stans, Austria; 4 Institute of Nutrition, University of Gießen, 35390 Gießen, Germany; 5 Institute of Primary Care, University of Zurich, 8000 Zurich, Switzerland; 6 Medbase St. Gallen Am Vadianplatz, 9000 St. Gallen, Switzerland; 7 Research Center Medical Humanities, Leopold-Franzens University of Innsbruck, 6020 Innsbruck, Austria

**Keywords:** running, marathon, female, motive, recreational athlete, endurance exercise, habit

## Abstract

Training for running events is fundamental for successful participation in various running events such as 10 km, half-marathon, marathon, or ultra-marathon distances. Training behaviors are likely based on runner motivations and social constraints, particularly for females. Participants completed a questionnaire following a cross-sectional approach, including questions on sociodemographics, general training behaviors, and periodization training strategies. The final sample included 245 participants (141 females, 104 males), mostly from Germany (72%), Austria (18%), and Switzerland (5%), with a median age of 39 years (IQR 17) and a BMI of 21.7 kg/m² (IQR 3.5). Males more often trained alone and independently, whereas females were most likely to follow an external resource (*p* = 0.037). Non-parametric ANOVA revealed significant training differences between sexes in daily training mileages and durations at each phase and stage (*p* < 0.05) as well as in weekly training mileages and durations for general basic training and race-specific training (*p* < 0.05). Critical sex differences in training behaviors may arise from physiological differences and social expectations, which may be related to the distances they prefer to race at as well as their motivations for running and racing. This study provides a wide overview of training behaviors for endurance runners or professionals guiding healthy running performance.

## 1. Introduction

Running has been well-established as a healthy physical activity for females and males across adulthood [1,2,3]. While many similar training habits exist between female and male endurance runners, a multitude of factors remain distinguishable between the sexes, including primarily physiological and social differences [4,5,6,7]. Numerous studies have investigated sex differences in the training behaviors of endurance runners [8,9,10,11,12,13,14,15]. To the best of the authors’ knowledge, no investigation has analyzed sex-related running training behaviors considering diverse distances, including 10 km (10 km) up to ultra-marathon (UM) distances in one study. 

Parallel to health benefits, frequent runners often follow various personal motivations for running, primarily for leisure or sports performance [3,4,16]. Females generally report higher running motives for psychological health reasons (body weight concern, a sense of belonging, life meaning, fulfillment/harmony), whereas males are mostly competition-focused [17]. Training provides the structure for progress in meeting the individual’s motivational goals [15,18] independent of sex. 

Considering the anatomical (e.g., anthropometric, hormonal, maximum muscular strength) [19,20] and societal (e.g., expectations, motives) differences between females and males [6,21], further sex differences in runner training behaviors may arise, including general training (training duration, external resource, other sports participation) and specific training measures (weekly frequency, weekly and daily mileages and durations, type of training from basic- to competition-specific, and various intensities) as well as the distances they prefer to run (10 km, half-marathon, marathon/UM) [9,15,17,18]. Regardless of the endurance runner’s motives, athletes typically adhere to periodization strategies when training for running events, including recognizable training phases that may unfold throughout the duration of up to a year before an event [22,23]. Different periods of training (regeneration and transitional period, main preparatory period, competition period) are followed to build and/or maximize beneficial training adaptations, mainly to improve the maximal volume of oxygen consumption in runners to maintain a faster pace for better finishing times [22,23,24].

Contrariwise to the sex differences in the training behaviors of endurance runners identified by the previous literature [8,9,10,11,12,13,14,15], similarities between the sexes are also commonly observed that appear contradictory [8,9,11,12,13,14]. However, this occurrence may be due to the previous study designs, the strict inclusion of pre-specified race distances, and the methodological heterogeneity between the studies at the population level [8,9,10,11,12,13,14,15]. Therefore, this study is the first aiming to assess the relationships between training behaviors of female and male recreational endurance runners over 10 km up to UM distances. The present investigation hypothesized that there are critical training differences between the sexes of recreational endurance runners of various distances (half-marathon, marathon/UM, 10 km).

## 2. Materials and Methods

The study protocol of the Nutrition and Running High Mileage (NURMI) Study [25] was approved by the ethics board of St. Gallen, Switzerland on 6 May 2015 (EKSG 14/145). The trial registration number is ISRCTN73074080 (retrospectively registered). Detailed information about the methods of the NURMI Study Step 2 has been described elsewhere [3,4,7,26,27,28,29,30]. 

The NURMI Study was conducted in three steps following a cross-sectional design. Endurance runners were mainly recruited from Austria, Germany, and Switzerland and were contacted primarily via social media, websites of the organizers of marathon events, online running communities, email lists, and runners’ magazines, as well as via magazines for health, nutrition and lifestyle, trade fairs on sports, plant-based nutrition and lifestyle, and through personal contacts. The characteristics of the subjects are presented in Table 1.

Participants completed an online survey within the NURMI Study Step 2 that was available from February 2015 to December 2015 in German and English at www.nurmi-study.com (accessed on 17 August 2022). Prior to completing the questionnaire on physical and psychological health (including the basic assignment to an area of sports, motivation and aim of running activities, and other sports to balance for running in order to better differentiate between predominant health, leisure, or sport performance-orientated approach to running), participants were provided with a written description of the procedures and participants gave their informed consent to take part in the study.

For complete participation in the study, the following inclusion criteria were required: (1) written informed consent, (2) at least 18 years of age, (3) questionnaire Step 2 completed, and (4) completion of at least a half-marathon distance running event within the past two years. However, as an additional criterion in this study, participants had to (5) select an event as the NURMI running event, including either a half-marathon (HM) or marathon (M) distance that they prepared for and subsequently finished in Step 3 (main NURMI Study: Step 2 linked to Step 3) [25]. 

An additional group of 91 highly motivated runners provided accurate and pragmatic answers with plenty of high-quality data and had not successfully participated in either a HM or M but in a 10 km race instead. To avoid an irreversible loss of these valuable data sets, those who met all inclusion criteria but named a 10 km race as their running event were kept as an additional race distance subgroup. 

To control for measures of (1) running activity (history, motivation, training, racing, etc.) and (2) diet, we included control questions in different survey sections. Incomplete, inconsistent, and conflicting data sets were excluded from the data analysis. Moreover, to control for a minimal status of health linked to a minimum level of fitness and further enhance the reliability of data sets, the body mass index (BMI) approach followed the World Health Organization (WHO) [31,32]. With a BMI ≥ 30, other health protective and weight loss strategies alongside running are foremost necessary to safely reduce body weight.

**Figure 1:**
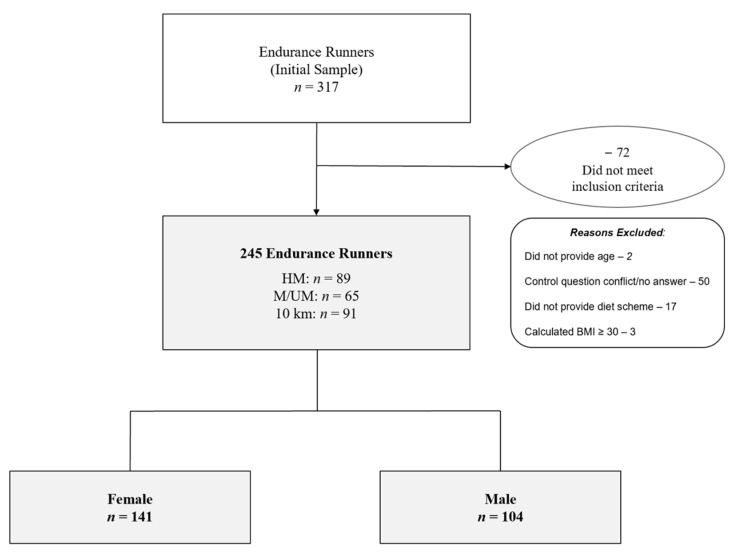
Enrollment and Categorization of Participants by Sex. BMI—body mass index. HM—half-marathon. M/UM—marathon/ultra-marathon. 10 km—10 kilometers.

Regarding the UM distance, the shortest UM reported was 50 km, and the longest was 160 km. Figure 1 shows the categorization of participants according to sex and race distance subgroups: HM, M/UM, and 10 km (M/UM data were pooled since the marathon distance is within an ultra-marathon). Additionally, the involved reader is kindly referred to the Part B publication for the subsequential linking of training behaviors with race performances [33].

Training behaviors of active female and male endurance running participants were described by the following items linked to sex: running motivations (health, leisure, sport performance); preferred time of day and preferred season for running activity (indoor vs. outdoor); training duration for the main event; external training resource followed (none, professional, other); participation in other sports activities to balance running (summer, winter); periodized running training (weekly frequency of training, daily and weekly mileages and durations of training (km, hours) related to phase and stage of training, respectively).

The statistical software R version 3.6.2 Core Team 2019 (R Foundation for Statistical Computing, Vienna, Austria) performed all statistical analyses. Exploratory analysis was performed by descriptive statistics (median and interquartile range (IQR); mean and standard deviation (SD)).

Significant differences in running activity (training behaviors) between race distance and sex were calculated by using a non-parametric ANOVA. Chi-square test (χ^2^; nominal scale) examined the association between variables, and the Wilcoxon test and/or Kruskal–Wallis test (ordinal and metric scale) approximated F distributions with ordinary least squares. 

Differences in weekly and daily training with the mileages of female and male runners are displayed as box plots.

The level of statistical significance was set at *p* ≤ 0.05.

## 3. Results

The survey was completed by 317 endurance runners. A total of 72 participants were excluded from the initial sample due to satisfying the exclusion criteria (i.e., three participants with a BMI ≥ 30) or disagreement with the inclusion criteria. After data clearance, a total of 141 (58%) females and 104 (42%) males (*n* = 245) comprised the final sample with a combined age of 39 (IQR 17) years, a body weight of 65 kg (IQR 14.2), and a BMI of 21.7 kg/m² (IQR 3.5) from several countries, including Austria (*n* = 44), Germany (*n* = 177), Switzerland (*n* = 13), and some other countries (*n* = 11: Belgium, Brazil, Canada, Italy, Luxemburg, Netherlands, Poland, Spain, United Kingdom). There were 154 NURMI-Runners (48% female) and 91 runners (74% female) competing at the 10 km distance. 

The female participants were significantly shorter (*p* < 0.001) and had a lower body weight (*p* < 0.001) and BMI (*p* < 0.001) than the males. Regarding the highest academic qualification achieved, participants reported a high school diploma or equivalent (53% female), an A Levels or equivalent (58% female), a university degree or higher (59% female), and some provided no answer (64% female). A significant difference (*p* = 0.044) was identified between the sexes in terms of exercise focus, where females were largely focused on leisure (57% female vs. 50% male), with a smaller proportion of females being sport performance-oriented (30% female vs. 44% male). Participants reported their marital status as single (32% of females vs. 20% of males), married or living with a partner (61% of females vs. 75% of males), or divorced/separated (7% of females vs. 5% of males). Characteristics of participants are further presented in Table 1, and more details on the characteristics of participants are provided in Part B of the sequenced paper [33]. 

**Table 1:** Characteristics of Endurance Runners Displayed by Sex.

		Total	Female	Male	Statistics
		100% (245)	58% (141)	42% (104)	
**Age (years)**		39 (IQR 17)	37 (IQR 16)	43 (IQR 18)	F_(1, 243)_ = 7.03 *p* = 0.009 ^†^
**Body Weight (kg)**		65 (IQR 14.2)	59.5 (IQR 10.9)	73 (IQR 11.9)	F_(1, 243)_ = 191.23 *p* < 0.001 ^‡^
**Height (m)**		1.7 (IQR 0.1)	1.7 (IQR 0.1)	1.8 (IQR 0.1)	F_(1, 243)_ = 228.04 *p* < 0.001 ^‡^
**BMI (kg/m^2^)**		21.7 (IQR 3.5)	20.9 (IQR 3.01)	22.8 (IQR 3.16)	F_(1, 243)_ = 28.72 *p* < 0.001 ^‡^
**Academic** **Qualification**	No qualification	<1% (1)	<1% (1)	/	χ^2^_(4)_ = 1.96 *p* = 0.744
	High school diploma/ Technical qualification/ GCSE or equivalent	34% (83)	31% (44)	38% (39)	
	A Levels or equivalent	22% (53)	22% (31)	21% (22)	
	University degree/ Graduate degree	34% (83)	35% (49)	33% (34)	
	No answer	10% (25)	11% (16)	9% (9)	
**Country of** **Residence**	Austria	18% (44)	11% (16)	27% (28)	χ^2^_(3)_ = 11.32 *p* = 0.010 *
	Germany	72% (177)	79% (112)	62% (65)	
	Switzerland	5% (13)	4% (6)	7% (7)	
	Other	4% (11)	5% (7)	4% (4)	
**Exercise Focus**	Health Leisure Sport performance	9% (23) 54% (133) 36% (89)	12% (17) 57% (81) 30% (43)	6% (6) 50% (52) 44% (46)	χ^2^_(2)_ = 6.24 *p* = 0.044 *
**Racing Distance**	HM M/UM 10 km	36% (89) 27% (65) 37% (91)	35% (49) 18% (25) 48% (67)	38% (40) 38% (40) 23% (24)	χ^2^_(2)_ = 19.55 *p* < 0.001 ^‡^
**Initial Running Motivation**	Health Leisure	44% (108) 56% (137)	46% (65) 54% (76)	41% (43) 59% (61)	χ^2^_(1)_ = 0.55 *p* = 0.459
**Current Running Motivation**	Health Leisure Sport performance	19% (47) 46% (113) 35% (85)	21% (30) 48% (67) 31% (44)	16% (17) 44% (46) 39% (41)	χ^2^_(2)_ = 2.06 *p* = 0.356

Note. *, †, or ‡ denote statistical significance at the levels *p* < 0.05, *p* < 0.01, or *p* < 0.001, respectively. Results are presented as percentage (%), total numbers, and median (IQR). χ^2^ statistic calculated by Pearson’s Chi-squared test and *F* statistic calculated by Kruskal–Wallis test. HM—half-marathon. M/UM—marathon/ultra-marathon. 10 km—10 kilometers.

No significant differences were found between the sexes for the initial running motivation (*p* = 0.459) or the current running motivation (*p* = 0.356). Seasonal running preferences did not differ between females and males, including the preferred indoor (*p* = 0.312) or outdoor running season (*p* = 0.727) and the ideal time of day for running, whether indoor (*p* = 0.419) or outdoor (*p* = 0.592). From the total sample, most participants preferred outdoor running (*n* = 145; 57% female) in the springtime and most often during the morning (*n* = 75; 57% female) but had no indoor running preference for a season (*n* = 178; 54% female) or time of day (*n* = 168; 54% female). 

**Figure 2:**
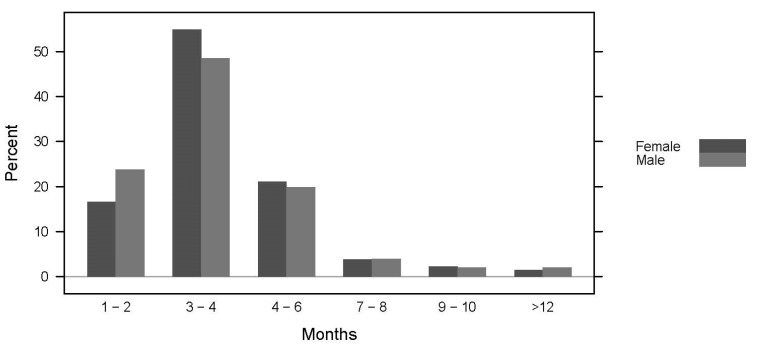
Sex-based differences in the prevalence of training duration (displayed in six categories) for main events. Data are presented by percentage.

Table 2 displays the general training behaviors for recreation runners for females and males. A significant difference was identified (*p* = 0.037) in which males were more likely to train alone (84% male vs. 71% female), whereas females were more likely to train under the direction of a professional (24% female vs. 12% male) or another resource (11% female vs. 4% male). Men were significantly more likely to participate in fell/trail running (*p* = 0.021) and ski-touring (*p* = 0.029); no additional sex-based participation differences were identified in other sports to balance running: cycling, swimming, rambling, triathlon, skiing, cross-country skiing, or snowboarding. No significant differences were observed between training duration for the main event and sex (*p* = 0.833) shown in Figure 2.

**Figure 3:**
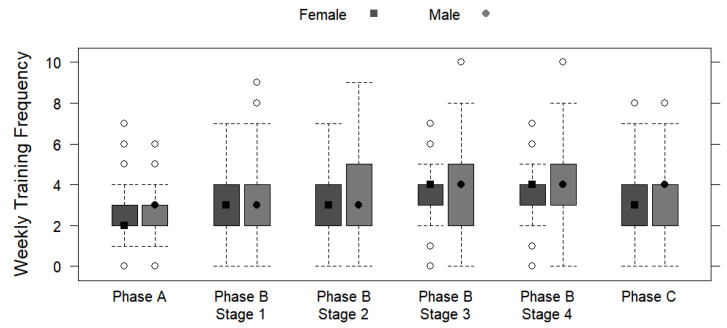
Box plots displaying sex differences in weekly training frequencies in different training periodization phases and stages.

**Figure 4:**
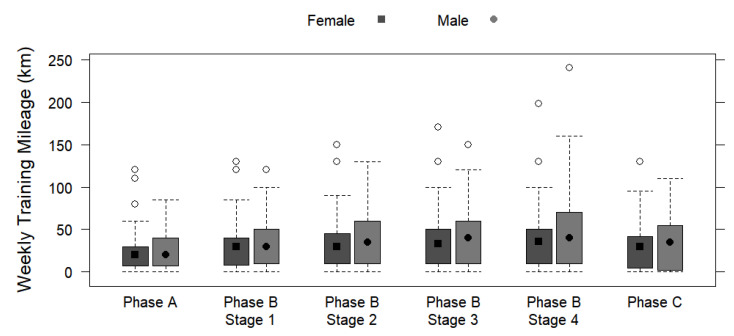
Box plots displaying sex differences in weekly training mileages (km) in different training periodization phases and stages.

**Figure 5:**
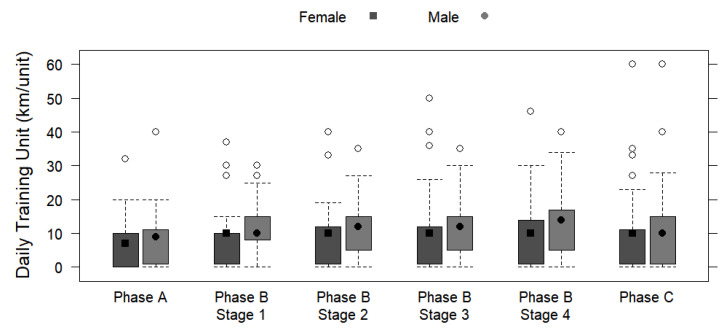
Box plots displaying sex differences in daily training unit (km/unit) in different training periodization phases and stages.

Table 3 displays the periodized training phases by sex, including the regeneration stage and transitional period (Phase A), the main preparatory period (Phase B), and the main competition period (Phase C) based on the weekly training frequencies and the weekly and daily training mileages and durations. No significant differences were observed for weekly training frequency and sex, regardless of the phase (*p* > 0.05). Preparatory Stage 4 showed the greatest average difference in weekly mileage (+11.4 km/week for males; *p* = 0.030) and duration (+1.71 h/day for males; *p* = 0.032). Significant differences were found between the sexes in terms of daily training mileage and daily training duration for every phase and stage (*p* < 0.05), with a highly significant difference identified in stage 1 (phase B) for daily training mileage (+2.94 km/day for males; *p* < 0.001) and duration (+0.14 h/day for males; *p* < 0.001). Figure 3, Figure 4 and Figure 5 depict the training mileages and weekly training frequency by sex based on the training periodization phases.

## 4. Discussion

The objective of this study was to investigate sex differences in training behavior among endurance runners; the present investigation was conducted to assess the relationships between training behaviors of female and male recreational runners from 10 km up to UM distances. The main findings were (1) female runners had a lower body weight and height and thus a lower BMI than the males; (2) a significant sex difference was found in main event racing distance; (3) significantly more females reported following an external training resource but no sex difference was found in the training duration for the main event; (4) weekly training frequencies were similar for females and males across periodization phases (including all stages of Phase B); (5) various discrepancies were identified between the sexes for weekly mileages and durations depending on each phase/stage; (6) males were significantly more active at all phases/stages considering daily mileage and duration; (7) males had a greater exercise focus on sport performance but no significant differences were found between sexes for the initial or current running motivations. While similarities exist between the training behaviors of female and male endurance runners [8,9,10,11,12,13,14,15], the results of the present investigation uphold the hypothesis that there are critical training differences between the sexes of recreational endurance runners of various distances (HM, M/UM, 10 km).

Based on the previous literature concerning female and male runner anthropometric differences [12], the present findings are consistent, indicating that females are generally smaller than their male counterparts. Moreover, the participants’ anthropometrics (body weight, height, BMI), especially of the females, highlight the general knowledge of recreational runners being a slim, fit, and healthy population [2,3,4,28,34]. All the while, only 3 of the initial participants (<0.01% of the sample) reported having obesity and were excluded from this study due to the required WHO-based BMI criteria [31,32]; thus, the typical healthy runner training and race preparation lifestyle is reflected by the participants’ BMI within this study [2,3,4,28,34]. 

A significant difference was found in this study in the proportions of race distance subgroups between the sexes, with the largest proportion of females being 10 km runners and a remarkably larger proportion of males being M or UM runners. Until recent years, female event participation in very long distances (HM, M, or UM) has been trailing that of males [35], and this result may be a reflection that this difference between the sexes has not been completely mitigated. Considering that the males were more heavily proportioned among the longer-distance racing subgroups (HM, M/UM), and that the previous literature has shown considerable variation in runner training behaviors based on this factor alone [15], it could be expected that there are significant critical sex differences in training behavior of endurance runners. 

Furthermore, it was found that significantly more females were training under the direction of a professional (whether a sport scientist, doctor of sports medicine, or trainer) or followed another resource rather than training alone and independently, which was more common among the males. This finding could be related to a generally higher level of health consciousness among females, while professional support is well-known to be beneficial for the health of runners and especially injury prevention [3,4,36]. In addition, while participation was similar in most other sports activities to balance running (cycling, swimming, rambling, triathlon, skiing, cross country skiing, and snowboarding), a sex difference for fell/trail running and ski-touring was detected. Regarding the finding of significantly more male participation in fell/trail running, a previous study found the opposite [37]; however, that report only analyzed runners of a specific trail race, and the current sample was more general [25]. Ski-touring, on the other hand, predominantly takes place in the backcountry with advanced-level terrain and increased avalanche risk; thus, one possible explanation for more male ski-touring participation may be a higher risk-seeking tendency among males [38]. Furthermore, no sex difference in the training duration for the main event was found, which could be considered the overarching periodization scheme [22]. This finding may be due to the fact that participating in an endurance running event requires a minimal preparatory period to safely complete each event, which is likely dependent upon the event race distance (e.g., 10 km, HM, M, or UM) [15,39].

Regarding the breakdown of the training periodization into three distinct phases (Phase A: Regeneration Stage and Transitional Period; Phase B: Preparatory Period; Phase C: Main Competition Period), no sex difference in the weekly frequencies of training was detected. Therefore, regardless of sex, participants trained regularly throughout each week and training phase, possibly due to the fact that regular exercise is well-accepted as being healthy and provides a plethora of benefits to each individual [34,40]. Within Phase A, no sex differences in weekly training mileage or duration were found, which is likely due to this particular phase having a main focus of recovery to avoid overloading as it comes directly after participating in the previous main event [41]. Therefore, it would be expected that the participants’ characteristics are somewhat unrelated to training in this phase, as the major goal of this phase is proper regeneration and a smooth transition to the main preparatory period [41]. However, significant differences between the sexes regarding their daily training mileages and daily training durations in Phase A were found, suggesting that males run a greater distance per day and also spend a greater amount of time running per day than females during this period. This finding is possibly due to the greater professional advice sought by females, as more resting is highly advisable for achieving maximal recovery during this period, which may be a concern of overuse injury or burnout for males who are seeking less professional support for training [41,42]. 

Within Phase B, there are four preparatory stages with distinct training characteristics. Weekly training mileages and durations were similar for females and males during Preparatory Stages 2 and 3, whereas significant sex differences were found for Stages 1 and 4. Considering the training similarities between the sexes in Stage 2 (build-up training, including specific basic training, and at a low-to-moderate intensity) and Stage 3 (intervals, pace, specific competition training, and moderate-to-high intensity), it appears that the training run distance and duration are unrelated to sex. Therefore, these particular types of weekly exercises may be integral to the training plans of endurance runners regardless of sex, which is consistent with previous reports [8,11]. For Stage 1 (general basic training at a low intensity mainly) and Stage 4 (test competition, race-specific training, and moderate-to-high intensity); however, the discrepancies between the sexes for weekly mileages and durations are likely related to the previously mentioned result of more females racing in the shorter distance (10 km) and more males racing in longer distances (M/UM) [15]. Furthermore, and in connection, both the daily training mileages and daily training durations showed significant differences between the sexes across all four stages of Phase B. Considering that runners of longer distances have been shown to run further mileage while training [15], these Phase B mileage differences between the sexes likely arise from the result that the males race over longer distances. Regarding Stage 1, the general basic training at a low intensity is dominant in the long-distance runner’s training plan (M/UM) [15,43,44], and the present results show that males run remarkably more during this period with an additional 2.94 km per day (+0.14 h). Previous research has found performance and health benefits to sustained loads of low-intensity training, including left ventricular hypertrophy and the consequential enhancements to stroke volume, maximal volume of oxygen consumption, and thus lower resting heart rate [45]. Indeed, moderate-to-high intensity training at Stage 4 would require extensively longer bouts of mileage and duration for the longer-distance runner to meet their adaptive training thresholds for building mitochondrial density and capillarization [44,46]. Previous research has not found any major link between sex and volume of oxygen consumption training adaptations [46]. For Stage 4, the specific training types (including test competition and race-specific training) are less likely to be related to beneficial physical adaptations but are rather mental preparations, which are highly important for the longer-distance runner, as very long distances (M/UM) require extensive durations of focus [47,48]. Therefore, the training durations (weekly or daily) would appear to be dependent upon the mileages (weekly or daily), as increases in the distance load require more time to complete with a specified running intensity [15].

Results for Phase C, which was considered the main competition period and included tapering and interim race stage/s, show that there was no sex difference for weekly training mileage or duration, which may be due to this phase including tapering, and the consequential drastic reduction in weekly training volume for both females and males compared to the prior phases [49]. However, considering that this phase also included interim race stage/s, which are likely related to the main event race distance, there may be a connection to the significant sex differences identified for daily training mileage and duration [15].

Exercise motivations may underlie much of the participatory behavior in endurance running [3,50]. The present results are in line with previous findings, that the male exercise focus is more sport performance-oriented than females and that females are more focused on health [50]. Regarding the finding of leisure as the female’s main exercise focus, this result is somewhat inconsistent with previous literature [50], which suggests that females are primarily health-focused. However, no significant sex difference was identified in terms of the participants’ current running motivations, which could partly explain why there were also some training similarities between the sexes within the current results. Therefore, it is possible that a small fraction of participants considered themselves to be athletes of another main sport instead of running. 

Causal ascriptions have been suggested as the foundation for developing a theory of motivation and emotion [51]. Therefore, it appears achievement-related projections, which are plausibly socio-bounded, have a major influence on the exercise focus differences among females and males [50,51]. In our sample, 96% of participants were from Austria, Germany, and Switzerland, suggesting a rather homogenous group in terms of social culture [52]. Those German-speaking countries are reported to have advanced economies even compared to other western nations, and the results indicate no sex difference in academic qualification, including a predominantly white-caucasian population with high-income levels, high quality of life, and a high life expectancy [52,53]. 

Comparable to other studies that follow a cross-sectional design, the presented results include some limitations that should be addressed when interpreting the findings. The sample size was relatively small, and considering the sex-based approach of the present investigation, there was an unequal distribution of sexes per se (58% females), and also within the race distances, including more females (48% vs. 23% of males) as 10 km runners. However, most of the participants were racing at the HM distance, marathon, or ultra-marathon distance. As the results are based on self-reporting survey methods, over- and under-reporting of answers are possible based on the sociological expectations of the participants’ cultures; however, control questions (e.g., race distance) were used to minimize this effect. Lastly, most participants were from Germany, Austria, and Switzerland, which may limit the interpretation of some of our findings to Western and European running cultures. 

While the present investigation includes some limitations, the results have the potential to add light to this specific gap in the current literature on sex differences in training behaviors of 10 km up to ultra distance recreational runners. Upon careful consideration, the findings may be particularly beneficial for athletic trainers, physical therapists, coaches, team physicians, exercise physiologists, and endurance runner athletes to refine the vital understanding of planning and applying an optimal, health-based training regimen for successfully running races. Furthermore, the underrepresentation of females in previous athletic samples studied regarding targeted sex-specific approaches and personalization to training and performance requirements is evident [5,6,22]. In addition, future studies should consider investigating sex differences of endurance runners by controlling for main event race distance associated with runner motives.

## 5. Conclusions

In summary, this is the first study aiming to investigate sex differences in training and race preparation behaviors of recreational runners of various distances, including HM, M/UM, and 10 km participants. The results indicate that: female runners are more likely to train with an external resource; male runners train at a higher volume (greater daily mileages and durations at every periodization phase; greater or null weekly mileages and durations at each phase); females exercise with more leisure focus; and males concentrate more on sports performance. It can be concluded that sex differences in training behaviors, which may originate from physiological differences and social expectations, can be related to the distances runners prefer to race and their motivations for running and racing. The results of this study provide a wide overview of the fundamental training behaviors of female and male recreational endurance runners of various distances (HM, M, UM, 10 km) that may be remarkably supportive for carefully designing and implementing a thorough training plan for endurance runners themselves or by their coaches, exercise physiologists, athletic trainers, physical therapists, sport scientists, or sports medicine doctors. 

## Figures and Tables

**Table 2:** General Training Behaviors, including Total Duration, Resource, and Other Sports by Sex.

		Total	Female	Male	Statistics
		100% (245)	58% (141)	42% (104)	
**Training Duration for Main Event**					
1–2 months 3–4 months 4–6 months 7–8 months 9–10 months >12 months		20% (46) 52% (122) 21% (48) 4% (9) 2% (5) 2% (4)	17% (22) 55% (73) 21% (28) 4% (5) 2% (3) 2% (2)	24% (24) 49% (49) 20% (20) 4% (4) 2% (2) 2% (2)	χ^2^_(2)_ = 2.12; *p* = 0.833
**Training Resource for Running Events**					
Alone and independently		76% (179)	71% (94)	84% (85)	χ^2^_(2)_ = 6.57; *p* = 0.037 *
Under the direction of a professional		15% (36)	24% (18)	12% (12)	
Other		8% (19)	11% (15)	4% (4)	
**Other Sports Activities to Balance for Running**					
*Summer*	Cycling	53% (130)	51% (72)	56% (58)	χ^2^_(1)_ = 0.57; *p* = 0.451
	Fell/Trail running	31% (75)	25% (35)	39% (40)	χ^2^_(1)_ = 5.32; *p* = 0.021 *
	Swimming	31% (75)	29% (41)	33% (34)	χ^2^_(1)_ = 0.39; *p* = 0.535
	Rambling	31% (75)	34% (47)	27% (28)	χ^2^_(1)_ = 1.13; *p* = 0.287
	Triathlon	19% (46)	17% (24)	21% (22)	χ^2^_(1)_ = 0.69; *p* = 0.407
*Winter*	Skiing	14% (34)	13% (18)	16% (16)	χ^2^_(1)_ = 0.35; *p* = 0.552
	Cross country skiing	11% (26)	10% (14)	12% (12)	χ^2^_(1)_ = 0.17; *p* = 0.681
	Snowboarding	7% (16)	7% (10)	6% (6)	χ^2^_(1)_ = 0.17; *p* = 0.682
	Ski-touring	4% (9)	1% (2)	7% (7)	χ^2^_(1)_ = 4.79; *p* = 0.029 *

Note. * denotes statistical significance at the level *p* < 0.05. Results are presented as percentage (%) and total numbers. χ^2^ statistic calculated by Pearson’s Chi-squared test and F statistic calculated by Kruskal–Wallis test.

**Table 3:** Periodization Training Behavior, including Frequency, Mileages, and Durations Displayed by Sex.

	Total	Female	Male	Statistics
	100% (245)	58% (141)	42% (104)	
**Phase A—Regeneration Stage and Transitional Period**				
Weekly training frequency Weekly training mileage (km) Weekly training duration (hours)	3 (IQR 1) 22.4 ± 18.9 1.21 ± 1.03	2 (IQR 1) 20.6 ± 19 1.12 ± 1.03	3 (IQR 1) 24.6 ± 18.7 1.33 ± 1.02	F_(1, 220)_ = 0.29; *p* = 0.590 F_(1, 220)_ = 3.77; *p* = 0.053 F_(1, 220)_ = 3.58; *p* = 0.060
Daily training mileage (km) Daily training duration (hours)	7.06 ± 5.86 0.26 ± 0.21	6.24 ± 5.33 0.23 ± 0.19	8.12 ± 6.36 0.30 ± 0.23	F_(1, 220)_ = 7.26; *p* = 0.008 ^†^ F_(1, 220)_ = 6.28; *p* = 0.013 *
**Phase B—Main Preparatory Period**				
***Preparatory Stage 1 (general basic training, mainly at low intensity)***				
Weekly training frequency Weekly training mileage (km) Weekly training duration (hours)	3 (IQR 2) 30.2 ± 24.6 4.63 ± 3.76	3 (IQR 2) 27.2 ± 23.1 4.16 ± 3.54	3 (IQR 2) 34.1 ± 25.9 5.22 ± 3.96	F_(1, 220)_ = 0.01; *p* = 0.931 F_(1, 220)_ = 4.40; *p* = 0.037 * F_(1, 220)_ = 4.67; *p* = 0.032 *
Daily training mileage (km) Daily training duration (hours)	8.74 ± 6.61 0.4 ± 0.3	7.46 ± 6.13 0.34 ± 0.28	10.4 ± 6.86 0.48 ± 0.31	F_(1, 220)_ = 14.93; *p* < 0.001 ^‡^ F_(1, 220)_ = 14.70; *p* < 0.001 ^‡^
***Preparatory Stage 2 (specific basic training, build-up training, low-to-moderate intensity)***				
Weekly training frequency Weekly training mileage (km) Weekly training duration (hours)	3 (IQR 2) 33.5 ± 27.5 4.82 ± 3.96	3 (IQR 2) 31 ± 26 4.46 ± 3.74	3 (IQR 3) 36.7 ± 29.2 5.28 ± 4.2	F_(1, 220)_ = 0.02; *p* = 0.902 F_(1, 220)_ = 2.15; *p* = 0.144 F_(1, 220)_ = 2.21; *p* = 0.138
Daily training mileage (km)	9.42 ± 7.29 0.41 ± 0.32	8.39 ± 7.09 0.36 ± 0.31	10.8 ± 7.37 0.47 ± 0.32	F_(1, 220)_ = 7.91; *p* = 0.005 ^†^ F_(1, 220)_ = 7.85; *p* = 0.006 ^†^
Daily training duration (hours)				
***Preparatory Stage 3 (competition training, intervals, pace, moderate-to-high intensity)***				
Weekly training frequency	4 (IQR 2) 37.1 ± 31.1 5.65 ± 4.73	4 (IQR 1) 34.2 ± 29.5 5.2 ± 4.48	4 (IQR 3) 40.9 ± 32.9 6.22 ± 5	F_(1, 220)_ = 0.04; *p* = 0.850 F_(1, 220)_ = 2.41; *p* = 0.122 F_(1, 220)_ = 2.57; *p* = 0.110
Weekly training mileage (km)				
Weekly training duration (hours)				
Daily training mileage (km)	9.98 ± 7.86 0.41 ± 0.32	9.01 ± 7.78 0.37 ± 0.32	11.24 ± 7.82 0.47 ± 0.32	F_(1, 220)_ = 8.37; *p* = 0.004 ^†^ F_(1, 220)_ = 8.20; *p* = 0.005 ^†^
Daily training duration (hours)				
***Preparatory Stage 4 (race-specific training, test competition, moderate-to-high intensity)***				
Weekly training frequency	4 (IQR 2) 39.5 ± 35.8 5.95 ± 5.39	4 (IQR 1) 34.6 ± 30.6 5.2 ± 4.6	4 (IQR 2) 46 ± 40.9 6.91 ± 6.15	F_(1, 220)_ = 1.49; *p* = 0.223 F_(1, 220)_ = 4.74; *p* = 0.030 * F_(1, 220)_ = 4.65; *p* = 0.032 *
Weekly training mileage (km)				
Weekly training duration (hours)				
Daily training mileage (km)	10.7 ± 8.31 0.5 ± 0.39	9.47 ± 7.93 0.45 ± 0.37	12.29 ± 8.56 0.58 ± 0.4	F_(1, 220)_ = 10.13; *p* = 0.002 ^†^ F_(1, 220)_ = 9.58; *p* = 0.002 ^†^
Daily training duration (hours)				
**Phase C—Competition Period (incl. tapering and interim race stages)**				
Weekly training frequency	3 (IQR 2) 32.2 ± 27.7 4.41 ± 3.8	3 (IQR 2) 28.8 (24.4) 3.95 (3.33)	4 (IQR 2) 36.6 (31.1) 5.01 (4.26)	F_(1, 220)_ = 3.30; *p* = 0.071 F_(1, 220)_ = 2.94; *p* = 0.088 F_(1, 220)_ = 2.48; *p* = 0.117
Weekly training mileage (km)				
Weekly training duration (hours)				
Daily training mileage (km)	9.35 ± 8.7 0.41 ± 0.37	8.46 ± 8.33 0.37 ± 0.36	10.5 ± 9.07 0.45 ± 0.39	F_(1, 220)_ = 5.21; *p* = 0.023 * F_(1, 220)_ = 4.47; *p* = 0.036 *
Daily training duration (hours)				

Note. *, †, or ‡ denote statistical significance at the levels *p* < 0.05, *p* < 0.01, or *p* < 0.001, respectively. Results are presented as median (IQR) and mean (SD). F statistic calculated by Kruskal–Wallis test. km—kilometer.

## Data Availability

The data sets generated during and/or analyzed during the current study are not publicly available but may be made available upon reasonable request. Subjects will receive a brief summary of the results of the NURMI Study if desired.
